# Online Detection of Impurities in Corn Deep-Bed Drying Process Utilizing Machine Vision

**DOI:** 10.3390/foods11244009

**Published:** 2022-12-11

**Authors:** Tao Li, Jinjie Tong, Muhua Liu, Mingyin Yao, Zhifeng Xiao, Chengjie Li

**Affiliations:** 1 College of Engineering, Jiangxi Agricultural University, Nanchang 330045, China; 2 Jiangxi Institute of Science and Technology Information, Nanchang 330000, China; 3 College of Engineering, South China Agricultural University, Guangzhou 510642, China; 4 Department of Chemical and Biological Engineering, University of British Columbia, Vancouver, BC V6T1Z3, Canada

**Keywords:** impurities content, corn, deep-bed drying, machine vision

## Abstract

Online detection of impurities content in the corn deep-bed drying process is the key technology to ensure stable operation and to provide data support for self-adapting control of drying equipment. In this study, an automatic approach to corn image acquisition, impurity classification and recognition, and impurities content detection based on machine vision technology are proposed. The multi-scale retinex with colour restore (MSRCR) algorithm is utilized to enhance the original image for eliminating the influence of noise. HSV (Hue, saturation, value) colour space parameter threshold is set for image segmentation, and the classification and recognition results are obtained combined with the morphological operation. The comprehensive evaluation index is adopted to quantitatively evaluate the test results. Online detection results show that the comprehensive evaluation index of broken corncobs, broken bracts, and crushed stones are 83.05%, 83.87%, and 87.43%, respectively. The proposed algorithm can quickly and effectively identify the impurities in corn images, providing technical support and a theoretical basis for monitoring impurities content in the corn deep-bed drying process.

## 1. Introduction

Mechanized grain production drives the large-scale and intensive planting of grain [1]. China’s grain output has ranked first in the world for many years. In 2022, China’s total summer grain output was 147.39 million tons, with an increase of 1.0% over 2021 [2]. However, the problem behind the high grain output that cannot be ignored is the huge grain loss at the front end during the consumption stage of its supply chain. Relevant research shows that, at the front end during the consumption stage of its supply chain, grain loss in the storage sector accounts for 21.08% of the total loss; on the other hand, the post-production loss rate of the rural grain circulation model is 23.61%, twice that of the urban model [3]. The low intelligent degree of grain drying and the poor level of drying equipment are dominant factors in the large grain loss, especially in centralized drying in rural areas. The core scientific problem behind the bottleneck of grain drying technology is open-loop control and the lack of reliable online detection devices for process elements. Research on the key technology of grain drying equipment mostly focuses on the sensing and attribute exploration of dried materials characteristics, such as moisture content, porosity, flow rate, and the system process parameters, such as ventilation resistance and tortuosity [4]. However, there are many impurities mixed into the freshly harvested grain due to the unsuitable parameter design of harvesting machinery, bad operating environment, weather conditions, and the lack of operator experience [5,6,7]. Although some impurities with large volume are removed by the filter before entering the dryer, a considerable amount of impurities are still fed into the dryer, together with the wet grain. The process of impurities entering with the grain into the dryer not only leads to the porosity change of the flowing layer, so as to change the tortuosity of the drying medium, the effective evaporation area coefficient, and the ventilation resistance of the drying phase interface [8], but also causes blockage or wear when contacting with some key components, such as the fan and reciprocating grain discharging device, thus affecting the service life of the machine [9]. The online detection of impurities in the grain drying process is not only of great significance to realize the self-adapting control of the drying process, but is also a necessary condition to ensure the stable operation of drying equipment. However, due to the complexity of many uncertain interference factors coexisting in the grain drying process, and considering factors such as test cost, measurement accuracy, material loss, and test period, little research has focused on the distribution characteristics of impurities in the grain drying system and their online detection, which greatly restricts the realization of high-quality and efficient grain drying.

Currently, the dryer operator generally obtains the impurity distribution condition in the dryer by the subjective observation of grain at the outlet. The standard weighing method [10] for impurity content measurement is generally less adopted due to its complex process and low efficiency. Because this lacks the data support of the real-time impurity content of each drying stage and tempering stage of the dryer, it is not only unable to find the potential faults of some key components of the dryer in time, but is also unable to provide feedback signals for the self-adapting controller. As an important branch of artificial intelligence, machine vision technology has been widely adopted in the research and application of intelligent agriculture because of its high efficiency, low labour intensity, and high intelligence in terms of path recognition [11], crop recognition [12], growth and pest detection [13], fruit and vegetable quality detection [14], and automatic picking [15]. The introduction of machine vision technology into the field of drying is expected to radiate new vitality for the development of this traditional discipline. Rezaei et al. [16] applied machine vision technology to measure the area shrinkage of potato slices during the drying process. Combined with an artificial neural network, he developed an algorithm for adjusting microwave power according to the detected moisture content. Khazaei et al. [17] applied machine vision technology to measure the shrinkage in the process of raisin production and controlled the grape drying process through a neural network. Li et al. [18] applied machine vision technology to obtain the changes in appearance and quality of shiitake mushrooms during drying and obtained the relative humidity of drying medium that was most conducive to the quality of shiitake mushrooms. It can be seen from the above research that, in the field of agricultural product drying, machine vision technology has been widely adopted to analyse the morphological characteristics of the materials to be dried. Therefore, the application of machine vision technology is expected to solve the problem of the online detection of impurities in the grain deep-bed drying process.

In some agricultural machinery and equipment, the exploration of machine vision technology to distinguish between objects and impurities is relatively mature. Wang et al. [19] first introduced cone beam CT technology into the detection of impurity content in grain and combined it with voxel point statistics to achieve fast and non-destructive calculation of grain impurity content. Liang et al. [20] creatively designed the segmentation line detection algorithm based on the K-means clustering algorithm and watershed algorithm, realized the accurate segmentation of complex grain particles, and designed a thousand-kernel weight measurement system for different grains. To solve the problem that domestic combine harvesters lack online monitoring devices for impurity content and crushing rate, Chen et al. [21] proposed paddy image collection based on machine vision, classification, and recognition of impurities and broken grains, which can quickly and effectively identify impurities and broken grains in paddy images, providing technical support for the online monitoring of paddy impurity content and crushing rate. The methods adopted in the above studies are mostly based on relatively complex algorithms, which are slow in detection speed and poor in anti-interference; additionally, the high hardware cost also limits its field application. However, what is quite different from the scenarios applied in the above research is that industrial-scale corn drying is a complex system with many interferences, such as heat, humidity, light, sound, dust, and mechanical vibration. The design of an online impurity detection device for the industrial-scale corn drying process should not only consider the design indicators of traditional sensors, such as range, resolution, and accuracy, but also fully consider the interference of changes in drying environment caused by changes in the objective conditions of the drying process. In addition, the design cost is also a factor that researchers should consider. However, no previous study has investigated the online detection method and device of impurities in the industrial-scale corn drying process, which dramatically limits the design and optimization of the controller for the grain dryer.

To summarize, information on the online detection method and device of the complex industrial-scale corn drying process using machine vision technology appears to be scant in the scientific literature. The objectives of this study are: (a) to propose a corn component classification and recognition algorithm based on colour eigenvalues; (b) to design an online impurity detection device for characterizing the operating conditions of drying equipment and providing adaptive control data feedback information. We present accurate classification and identification methods and propose a low-cost detection device of impurities during drying that enhances the intelligence level of industrial-scale drying equipment. Additionally, this study provides technical support for the fault diagnosis of the drying process and the designing of high-quality and efficient grain drying equipment.

## 2. Materials and Methods

### 2.1. Image Acquisition System

**Figure 1:**
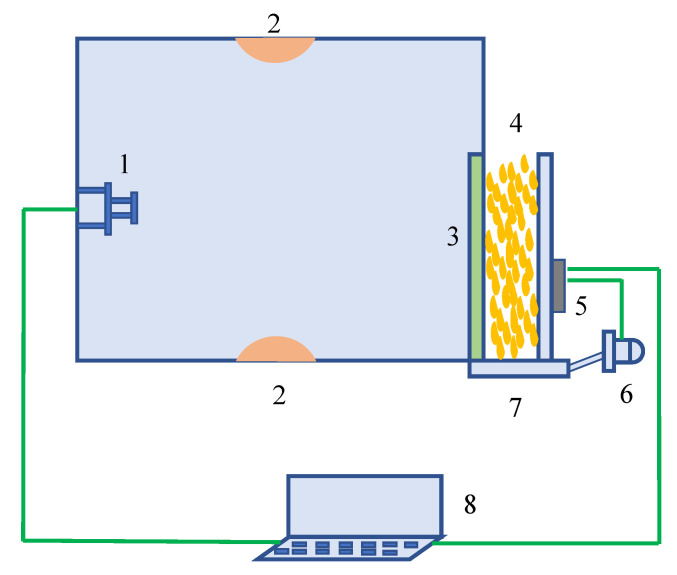
Schematic diagram of online detection device for impurities. Note: 3, transparent acrylic plate. 1, image sensor. 2, LED light. 4, Corn. 5, Main controller. 6, Drive motor. 7, Baffle. 8, Computer terminal.

The image acquisition device of the online impurity detection system for the corn deep-bed drying process was mainly composed of the image acquisition unit, the data transmission unit, the host computer unit, and the motion control unit, as shown in Figure 1. The image sensor was a CMOS sensor (OV7725, Howell Technology Co., Ltd., Zhengzhou, China) with high integration, low power consumption, low illumination adaptability, resistance to temperature change, and low cost. The sensor was equipped with a camera lens with a focal length of 3.6 mm (field angle of view was 78°) to collect 8-bit JPG format RGB images with an acquisition resolution of 600 × 480 pixels. The data transmission module was an nRF24L01 + 2.4 G wireless transmission module, which wirelessly transmitted the collected image to the host computer for image recognition and processing. Furthermore, the supporting software was compiled for the upper computer to identify and classify various impurities while displaying the collected images in real time and calculating the impurity content. The motion control unit consisted of a lower computer STM32 and a motor, which mainly completed the opening and closing operation of the baffle at the bottom of the sampling tank.

The image acquisition system was installed between the grain inlet and the conveyor at the top of the drying tower or between the outlet of the discharging hopper and the hoist to collect corn (4) falling into the grain sampling tank. After the computer terminal (8) received the filling signal from the material level sensor, the LED light (2) was turned on and a corn image was acquired through the image sensor (1) so as to complete the image display, recognition, and processing. After image processing was completed, the computer sent a grain discharging signal to the main controller (5) of the motion control unit to the drive motor (6) to open the baffle (7). After all tested corn were discharged, the baffle was closed, and the complete test process was finished.

### 2.2. Image Acquisition and Processing

#### 2.2.1. Image Acquisition

**Figure 2:**
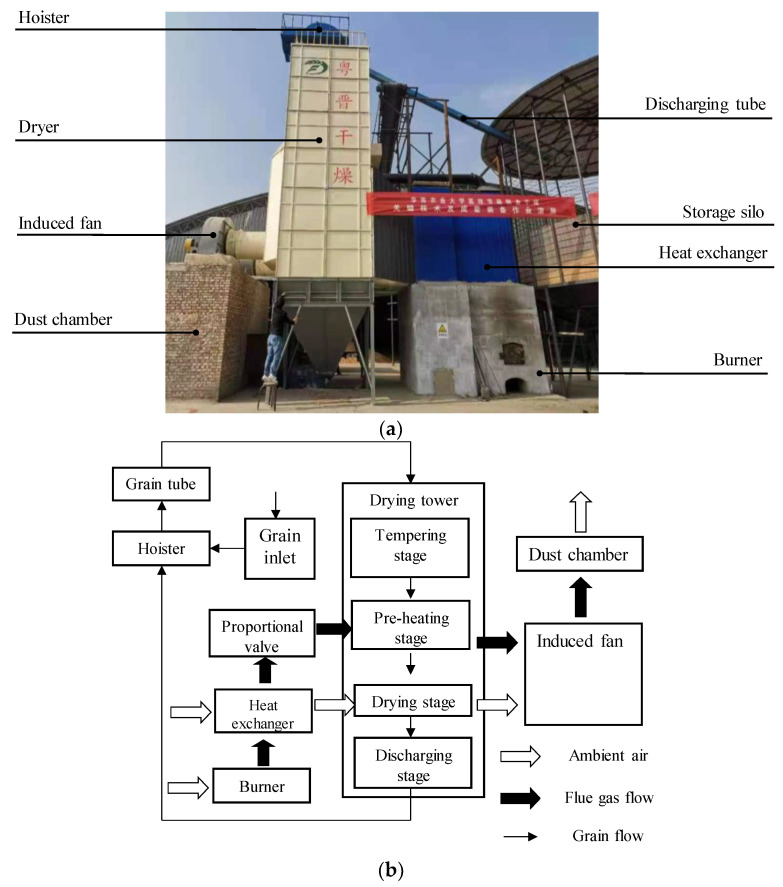
Circulation dryer (**a**) and its structure schematic (**b**).

The image acquisition was conducted at Zhencheng Farm in Xinzhou Shanxi Province, China. The dryer in operation was a 5HP-50 double tower circulation counter-flow corn dryer designed by our laboratory. The circulation counter-flow dryer and its structural principle are shown in Figure 2. More details about the self-developed dryer can be found in the related study [22] conducted by our team. The material to be dried was Changcheng 799# fresh corn with an average initial moisture content of 27.9% w.b. The image acquisition system depicted in Figure 1 was installed between the outlet of the discharge hopper and the hoist. Before the experiment, the working performance of the dryer was adjusted to ensure that the dryer was in a stable state during the experiment. The test sensor was calibrated and correctly installed. According to the operating system of the dryer, the elevator started to fill the drying chamber with fresh corn, and the grain discharging capacity and the corn initial moisture content were recorded. The fan and grain discharging mechanism were started successively through the control system, and the appropriate frequency of the grain discharging motor and the limit value of the drying air over temperature alarm were set. According to the test requirements, the main parameters, such as drying time, inlet air temperature, outlet air temperature and humidity, grain temperature and moisture content, and ambient air temperature and humidity during the drying process, were collected through the data collection centre until the end of the circulating flow process. When corn was dried to the target moisture content, the grain discharging pipe was used to transport dried corn to the grain storage bin for storage, and finally the whole drying process was completed. During the 9 h drying process, 5 images were randomly collected every 20 min, and the operation process was tracked continuously for 5 days. A total of 675 images were obtained, of which 20 images were randomly selected as samples.

#### 2.2.2. Image Recognition Process

**Figure 3:**
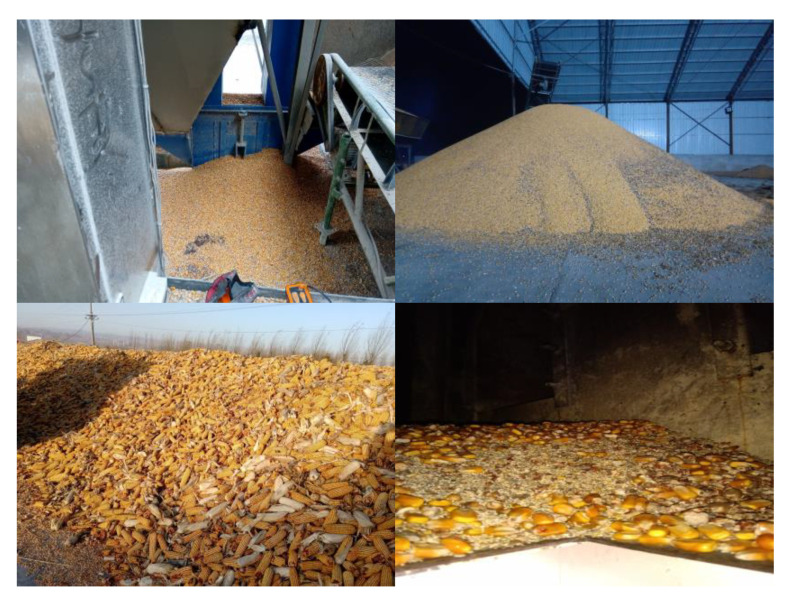
Main impurities mixed with corn to be dried.

The impurities in grain mainly include organic impurities belonging to the crop that were not filtered out in the pre-treatment process of harvest and threshing, such as straw and chaff, as well as inorganic impurities mixed in the process of cleaning and transportation, such as crushed stones. Figure 3 shows the corn to be dried photographed at the drying site. From Figure 3, the impurities in the corn mainly included broken corncobs, broken bracts, and crushed stones. Therefore, this study adopted machine vision technology to study the impurities recognition of the above three impurities.

**Figure 4:**
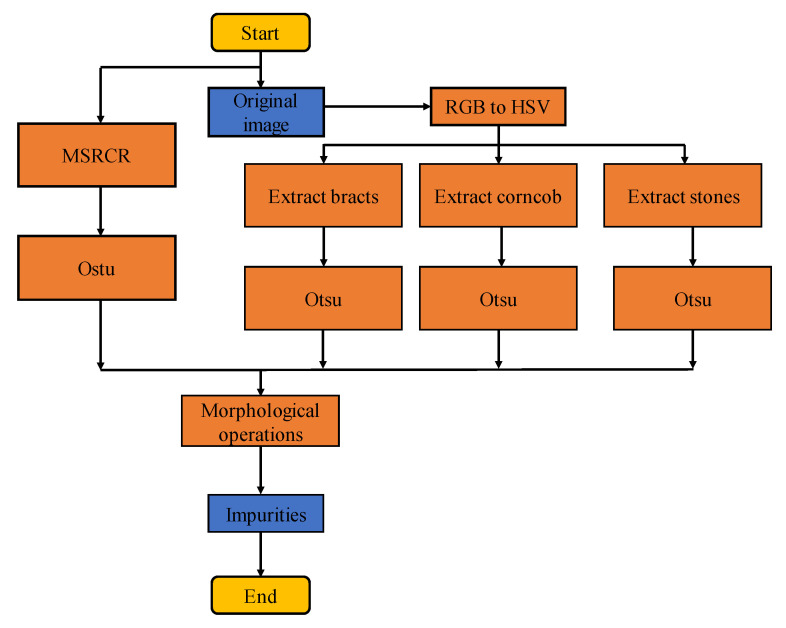
Image processing flow chart.

In this study, the essence of impurity recognition is to distinguish impurities from corn. Therefore, the foreground of the obtained image should be impurities, and the background should be corn. To background the corn seeds, the original image was transformed into an appropriate colour space to extract the attribute parameters that can characterize the classification. Through the statistics of the values from each channel component of impurities, different thresholds were set to roughly extract various impurities. Then, multi-scale retinex with a colour restore (MSRCR) algorithm was adopted to enhance the original image for compensation and elimination [23]. A maximum interclass variance method (Otsu) was used to binarize the original image and the roughly extracted impurity image, and then various impurities were identified through a series of morphological operations [24]. The flow chart of image processing is shown in Figure 4.

#### 2.2.3. Colour Feature Extraction

**Figure 5:**
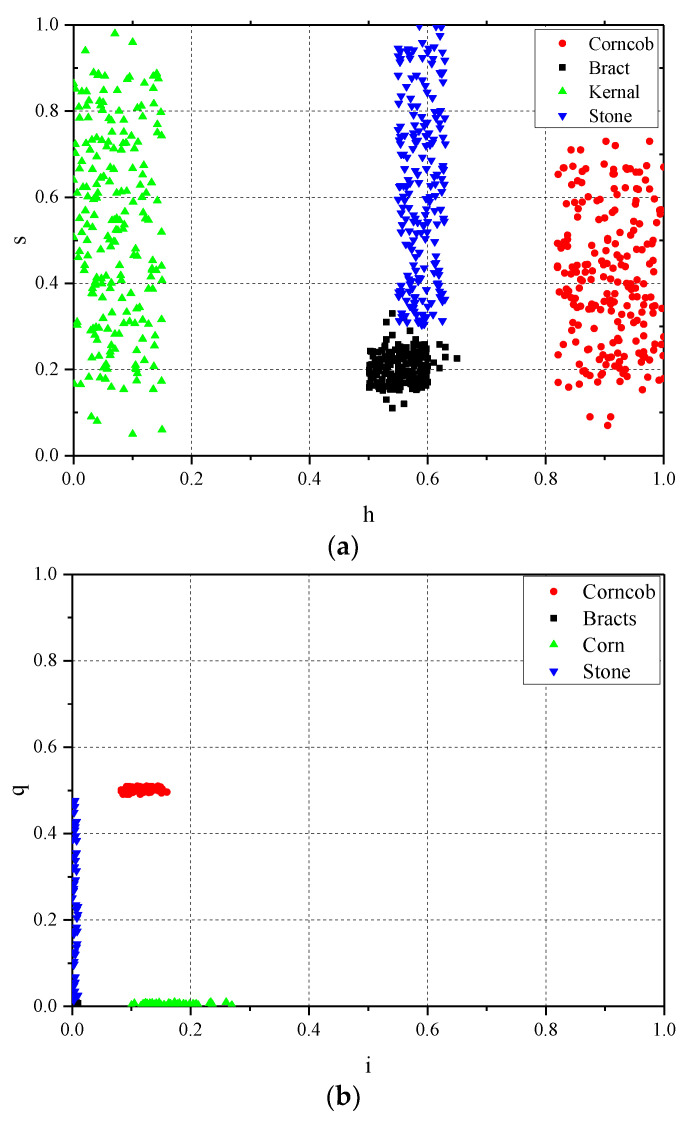

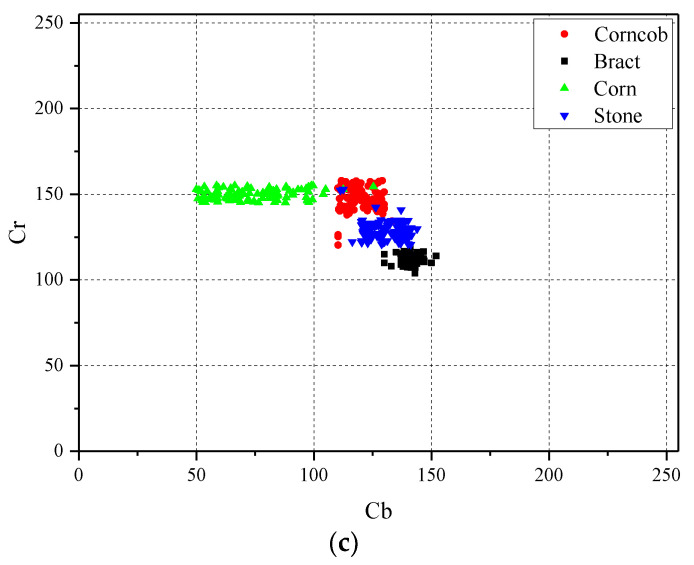
Pixel intensity values of each channel of the image in different colour spaces: (**a**) statistical chart of H and s range in HSV space, (**b**) statistical chart of i and q range in NTSC space, (**c**) statistical chart of Cb and Cr range in YCbCr space.

According to the main components of corn impurities shown in Figure 3 and their complex colour features, this study combined the colour features and shape features in HSV, NTSC, and YCbCr colour space, which were more suitable for colour expression in machine vision, to classify and identify the impurities in corn. After manually intercepting the internal areas of some broken corncobs, broken bracts, and broken stones in the sample image, and making statistics on the pixel component parameters in the area, the statistical results of the distribution range of colour characteristic parameters of various complete impurities were obtained, as shown in Figure 5. It should be mentioned that Figure 5 shows the intensity of each pixel of the image in three colour spaces after the channel related to the illumination intensity was removed. Thus, the influence of illumination on the image was excluded. It can be seen from the figure that the values of each channel in the NTSC and YCbCr colour space overlapped partially, which was not conducive to classification and recognition. Therefore, this study set appropriate thresholds for the H and S channels in HSV colour space, and identified impurities in combination with shape features.

#### 2.2.4. Quantitative Evaluation Model of Identification Results

Referring to the method of the online monitoring of soybean mechanized harvest quality proposed by Chen [15], the performance of the online impurity detection device in the corn deep-bed drying process developed in this paper was quantitatively evaluated by using precision ratio (*P_a_*) and recall ratio (*R_a_*). The precision ratio represented the ability of the system to identify impurities and corn kernel, while the recall rate represented the accuracy of the system to identify impurities, which are calculated by Equations (1) and (2):

(1) $$P_{a}=T_{p}/(T_{p}+F_{p})\times 100\%$$

(2) $$R_{a}=T_{p}/(T_{p}+F_{n})\times 100\%$$

where *T_p_* is the number of correctly identified pixels, *F_p_* is the number of incorrectly identified pixels, and *F_n_* is the number of missing identified pixels, which are obtained by manual annotation. In order to obtain the comprehensive detection performance, comprehensive evaluation index *F*_1_ is adopted to consider the overall detection index, and its calculation formula is as follows:

(3) $$F_{1}=2P_{a}R_{a}/(P_{a}+R_{a})\times 100\%$$

#### 2.2.5. Quantitative Model of Impurity Content

The traditional method to detect the grain impurity content is the standard weighing method [10]. After cleaning the grain samples, the impurities are screened out and weighed. The impurity content is obtained by calculating the ratio of impurities weight to grain weight. This method is time-consuming and cumbersome, and it is not suitable for online detection. Utilizing the online impurity detection system designed in this study, a quantitative model of impurity content based on machine vision was proposed. The calculation formula is as:

(4) $$P_{z}=\rho_{z}T_{z}/(\rho_{z}T_{z}+\rho_{w}T_{w})\times 100\%$$

where *P_z_* is the impurity content, *ρ_z_* is the average quality of impurities per 1000 pixels of image, *T_z_* is the number of impurity pixels recognized by the detection system, *ρ_w_* is the average quality of corn per 1000 pixels of the image, and *T_w_* is the number of corn pixels recognized by the detection system. *ρ_w_* and *ρ_z_* are 7.31 × 10^−3^ mg/pixel and 1.05 × 10^−3^ mg/pixel, respectively, which are obtained by manual annotation

## 3. Results and Discussion

### 3.1. Image Preprocessing Results

**Figure 6:**
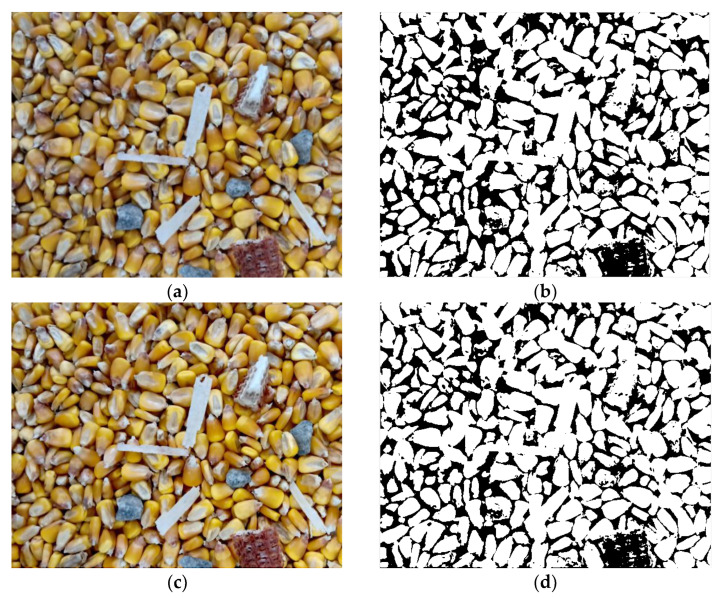
Image pre-processing results. (**a**) Original image, (**b**) Binary image of original image, (**c**) Image enhancement using MSRCR, (**d**) Binary image of enhanced image.

Grain deep-bed drying is a multiple disturbance process under the complex environment of high humidity, high dust, and light change [25,26]. Under this condition, if the acquired image is recognized without pre-processing, an error of impurity recognition may be introduced by noise interference. Therefore, it is necessary to enhance the acquired original image for compensation and elimination. Figure 6b is an image obtained by binarization of the original Figure 6a using the Otsu method. Due to the dark light on the left and upper sides of the original image and the distribution of some dust, the corn was not correctly binary divided into foreground. Moreover, due to the stacking among corn kernels in the original image, the boundary was not clear, which caused some grain adhesion in the binary image. After the original image was enhanced using the MSRCR method (Figure 6c), and then binarized using the Otsu method, Figure 6d was obtained. Compared with Figure 6b, the enhanced binary image not only had more accurate recognition on the left and upper sides of the image, but also had a certain improvement in the resolution of the whole image. Thus, it was more suitable for subsequent impurities classification and recognition. Compared with some improved watershed algorithms, such as those adopted in Zhang’s research [27], the classical Otsu method has higher computational efficiency.

### 3.2. Classification and Recognition Results

**Figure 7:**
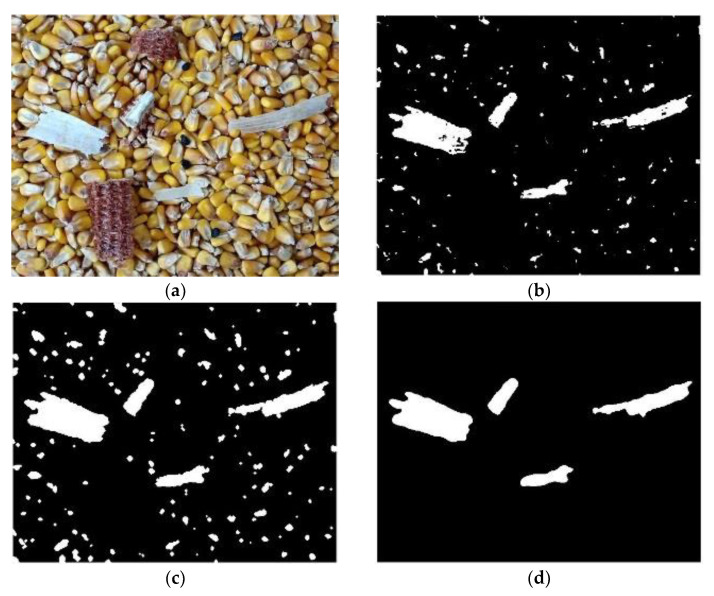
Identification of broken bracts. (**a**) Original image 2 of corn, (**b**) Coarse extraction of broken bracts impurities, (**c**) Filling the hole, (**d**) Broken bracts impurities.

From the statistical results of the h and s values of broken bracts, it was found that the h-value range of pixels on broken bracts was [0.48, 0.6], and the s-value range was [0.15, 0.25]. Therefore, the threshold range of the H and S channels were respectively set as [0.48, 0.6] and [0.15, 0.25] to extract the broken bract impurities, which ensured that all the broken bract impurities were correctly extracted. The extracted image and the binary image obtained by pre-processing were then calculated using the ‘AND’ operation to obtain the rough extracted image (Figure 7b). Compared with the binary image obtained by pre-processing, the interference of some corn grains in the image was eliminated, but there were many loopholes in the identified bracts, and the radicle at the tip of the grains still had more interference to the recognition. The ‘OPEN’ operation was a process of corrosion before expansion, which ensured a smooth boundary without changing the area. Therefore, the method of ‘OPEN’ operation and hole filling was adopted to eliminate small objects, separate the objects from the thin ones, smooth the larger objects, and fill the internal loopholes of impurities (Figure 7c). Finally, the impurities of broken bracts were further extracted by setting the threshold range of the area parameters. It was found that the area of broken bracts was more than 4500 pixels. Therefore, after setting the area threshold to 4500, the connected domain less than 4500 pixels was filled with black, and that greater than 4500 pixels was reserved to eliminate the radicle of the tip. Thus, the broken bract impurity shown in Figure 7d was obtained. Comparing Figure 7a,d, the extracted broken bracts were basically consistent with those in the original image in size and contour.

**Figure 8:**
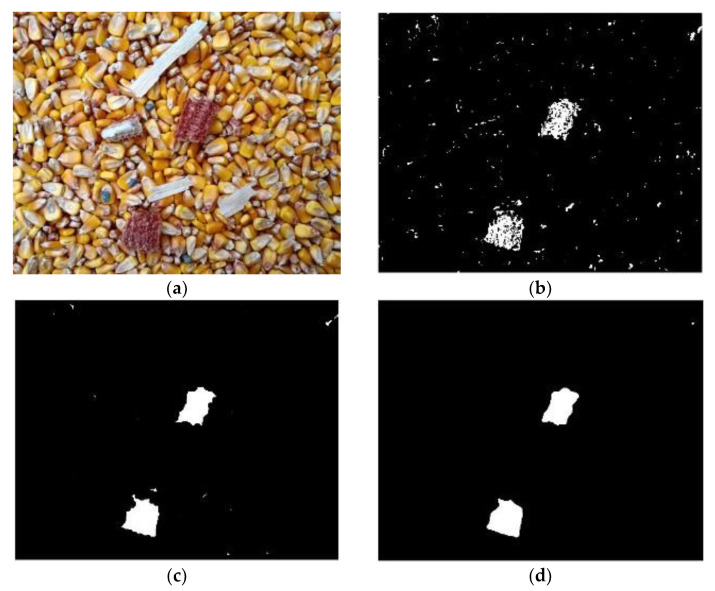
Identification of broken corncobs. (**a**) Original image 3 of corn, (**b**) Coarse extraction of broken corncobs impurities, (**c**) Close operation, (**d**) Broken corncobs impurities.

The broken corncob included the outer red face upward pieces and the inner white face upward pieces. Because the pixel intensity of the interior of the corncob was the same as the broken bracts, they can be identified together in the process of identifying the broken bracts. The h-value range of pixels on the outer red face upward pieces was [0.8, 1], and the s-value range was [0.06, 0.7]. Therefore, the threshold range of H and S channels were respectively set as [0.8, 1] and [0.06, 0.7] to extract the broken corncobs. The crude extraction diagram shown in Figure 8b was obtained by adopting the same method as the crude extraction of broken bracts. The method of ‘CLOSE’ operation and hole filling were then used to eliminate small objects, bridge small cracks, and fill the holes in broken corncobs. Finally, the median filtering method was utilized to remove the broken points and to optimize the edges for the purpose of obtaining the broken bract impurities, as shown in Figure 8d. It was easier to identify the broken corncobs than the broken bracts because the outline of a broken corncob was a rectangle with a uniform shape. Comparing Figure 8a and Figure 8d, it can be seen that the broken corncob had been well identified.

**Figure 9:**
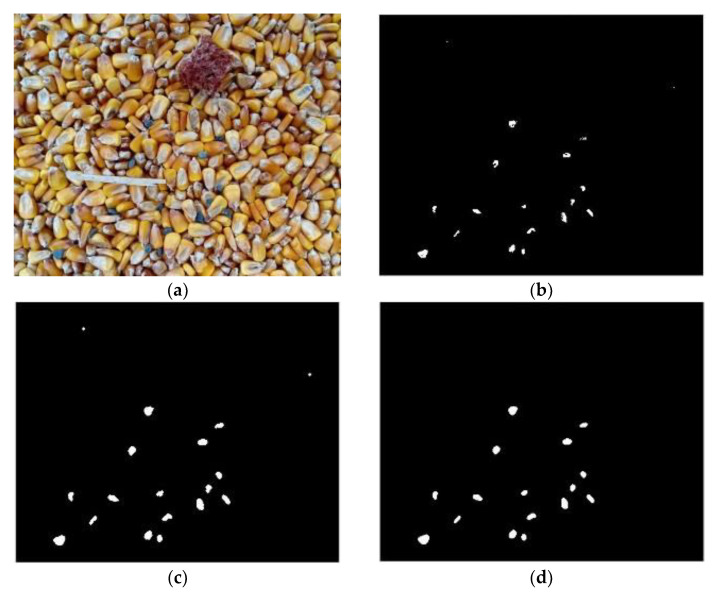
Identification of crushed stones. (**a**) Original image 4 of corn, (**b**) Coarse extraction of crushed stones impurities, (**c**) Expanding, (**d**) Crushed stones impurities.

Although the crushed stone has a small amount and small volume, the occurrence of these impurities often affects the accuracy of the online corn moisture content detector, and might seriously affect the operation of some key components of the dryer. Therefore, it is necessary to identify the crushed stone impurities. The h-value range of pixels on the crushed stones was [0.55, 0.65], and the s-value range was [0.28, 1]. Therefore, the threshold range of the H and S channels were respectively set as [0.55, 0.65] and [0.28, 1] to extract the crushed stones. After the extracted image and the binary image obtained by pre-processing were calculated using the ‘AND’ operation, the incomplete shape of some crushed stones was found (Figure 9b), and it was necessary to restore their original shape. After the morphological expansion operation was adopted for Figure 9b, the ‘AND’ operation was carried out with the binary graph of the original image to obtain the crushed stones with restored shape. It was found that the area of crushed stones was less than 400 pixels. Therefore, after setting the area threshold to 400, the connected domain greater than 400 pixels was filled with black, and less than 400 pixels were reserved to obtain the crushed stones, as shown in Figure 9d. Compared with Figure 9a,d, it can be seen that all crushed stones were basically well identified.

### 3.3. Impurity Identification Results

**Figure 10:**
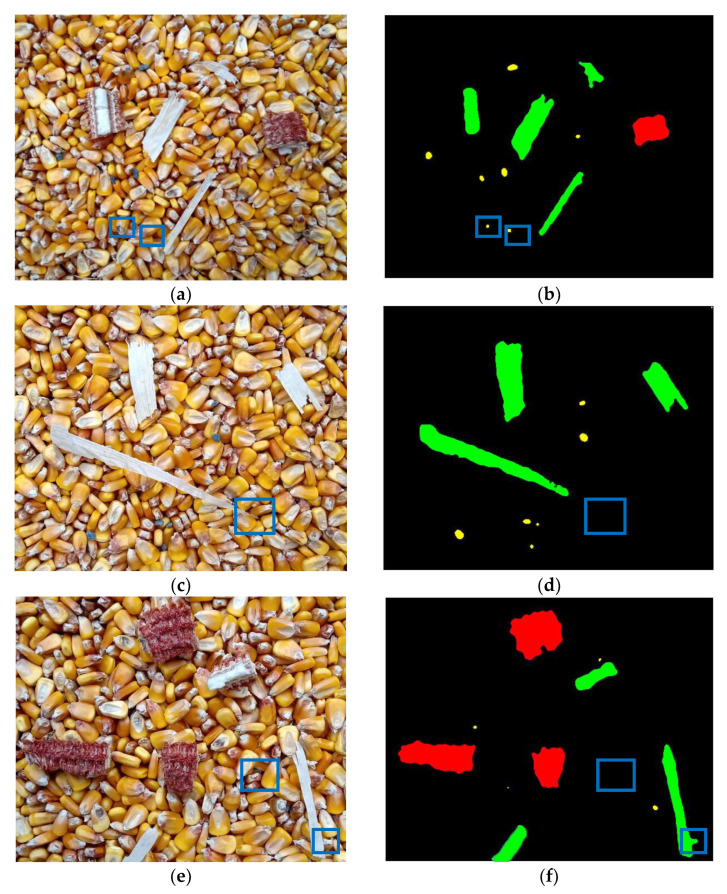
Recognition results of corn images. (**a**) Experimental original image 1, (**b**) Identification result 1, (**c**) Experimental original image 2, (**d**) Identification result 2, (**e**) Experimental original image 3, (**f**) Identification result 3.

Figure 10 shows three images randomly selected from the obtained corn images and their corresponding impurities recognition results. In general, impurities in the corn had been effectively identified, but there was also some wrong or missing identification. Because there was a rotten phenomenon in the radicle of some corn kernels, its colour component and shape were similar to those of crushed stones, so it was wrongly identified as crushed stones (Figure 10a,b). The colour component of the tip of a broken bract was similar to that of the root of the corn kernels, and its pixel area was small, so it was classified as the root of a corn kernel and missed identification (Figure 10c,d). There was obvious adhesion between broken bracts and corn kernels, which resulted in the wrong identification being attributed due to the lack of a clear boundary between broken bracts and kernels. In addition, the overlap between some crushed stones and corn kernels resulted in a small exposure area, which caused the overlap with background and missing identification (Figure 10e,f). Therefore, in order to consider the accuracy of the proposed algorithm for impurity identification, the quantitative method should be utilized to comprehensively evaluate the identification results.

### 3.4. Quantitative Evaluation of Identification Results

The detection system in this study was utilized for the recognition test using 100 corn sample images that were randomly selected in the samples, and the quantitative evaluation statistical results shown in Table 1 were obtained.

From the quantitative evaluation results, it can be seen that the *F*_1_ of a broken corncob reached 83.05%, indicating that its shape and size were basically recognized accurately. In image processing, to smooth the edge and to corrode the broken points, the burrs on the edge of the broken corncob were filtered, which caused errors in detection. In addition, some broken corncobs were rotten, resulting in colour changes and missing identification. The *F*_1_ value of broken bracts recognition reached 83.87%, indicating that most broken bracts were recognized effectively. Because the colour of the tip of broken bracts was similar to the root of corn kernels, and the boundary between some broken bracts and the tip of corn kernels was not obvious, some recognition errors were caused. The *F*_1_ value of crushed stone recognition reached 87.43%, which was the highest among all impurities. This is due to the obvious shape and colour difference among crushed stones, corn kernels, and other impurities. The reason for some recognition errors was that a small amount of corn kernel radicle decayed, and its colour and shape were similar to crushed stones, so it was wrongly recognized as crushed stones. It can be concluded that the decay phenomenon in the corn pile to be dried is the main reason for the identification error. The centralized deep-bed drying of grain generally occurs a short time after harvest. On the premise of no long-distance transportation and no long-term storage, there is little or even no decay. Therefore, it is feasible to utilize the detection system in this study to identify the corn impurities. The research results are close to Chen’s research results [5] on the identification of various impurities in paddy images, including stems, branches, and broken seeds.

### 3.5. Field Test Results

**Figure 11:**
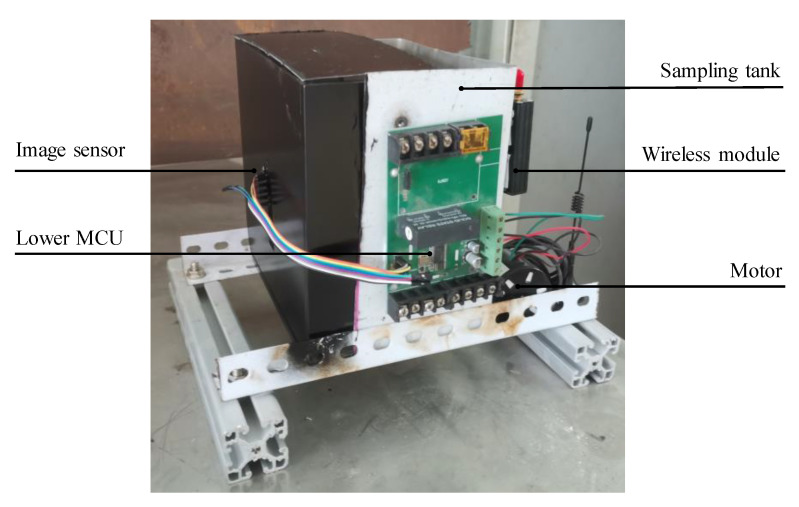
Prototype of online impurities detection device in the corn deep-bed drying process.

**Figure 12:**
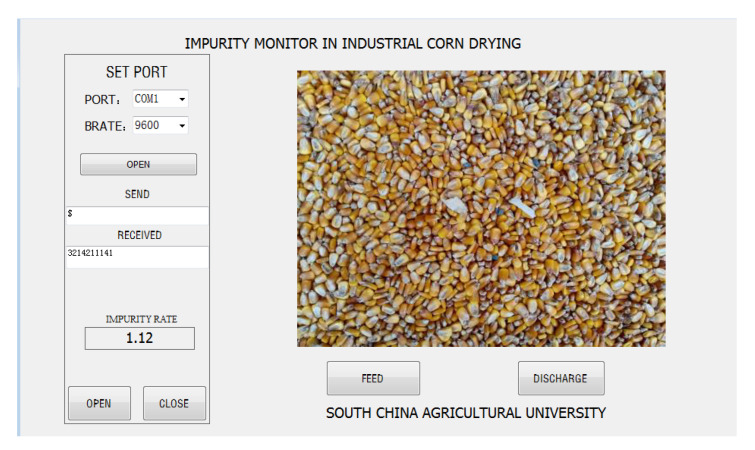
Host computer interface.

**Figure 13:**
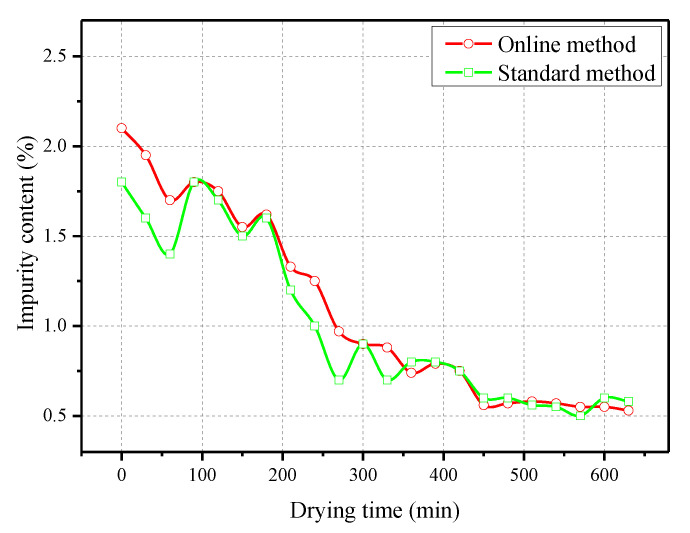
Impurity content measured by standard method and online method in the corn drying process.

The prototype and host computer interface of the online impurities detection device in the corn deep-bed drying process are shown in Figure 11 and Figure 12, respectively. The prototype was installed between the outlet of the discharge hopper and the elevator to automatically record the corn impurity content in the drying process, and the sampling period was 30 min. Meanwhile, some corn cobs were manually sampled, and the impurity content was detected according to the standard method [10] for comparison. The test results are shown in Figure 13.

From Figure 13, the trend of impurity content measured by the standard method and online method conformed to the actual drying process. During the continuous corn flowing process in the dryer, some impurities were discharged from the dryer under the negative pressure of the fan, resulting in the continuous reduction of impurity content in the drying process, which finally stabilized at around 0.5%. In the initial stage of drying, the offline measured value was basically slightly less than the online value, and the two gradually coincided in the later stage. The average of impurity content measured online and offline during the whole drying process were 1.09% and 1.01%, respectively, and the average error was 0.08%. The field test results show that the detection efficiency is greatly improved compared with the standard weighing method [10]. Therefore, the detection system designed in this paper can be used as an effective method of online detection of impurities in the corn deep bed drying process for the purpose of providing technical support for equipment condition detection and self-adapting control.

## 4. Conclusions

1. A corn deep-bed drying process image acquisition system was developed based on machine vision technology, and the supporting software was also compiled. The system can collect clear corn images in real-time to segment and identify each component of the image and calculate the real-time impurity content in the drying process. The result provides technical support for the accurate online detection of moisture and improvement of flow state during the deep bed drying of corn, and lays a theoretical foundation for adaptive control of the drying process.
2. The MSRCR algorithm was used to segment the collected corn image. Each independent closed area of the image was traversed, and the closed areas were classified by setting the HSV colour space parameter threshold to identify broken corncobs, broken bracts, and crushed stones in corn samples. The precision rates of broken corncobs, broken bracts, and broken stones were 83.15%, 84.61%, and 87.62%, respectively. The recall rates of the three impurities were 82.96%, 83.14%, and 87.27%, respectively. The comprehensive evaluation values of the three impurities were 83.05%, 83.87%, and 87.43%, respectively.
3. The average error between the online detection results of impurity content using the detection device and the manual detection results in the corn deep-bed drying process was 0.08%, which indicated that the system can accurately analyse the impurity content in the corn drying process. Therefore, the system can be used as an effective method for the online detection of impurities in the corn deep-bed drying process.

## Figures and Tables

**Table 1:** Quantitative evaluation statistical results of impurities identification.

Category of Impurities	P_a_/%	R_a_/%	F_1_/%
Broken corncob	83.15	82.96	83.05
Broken bracts	84.61	83.14	83.87
Crushed stones	87.62	87.24	87.43

## Data Availability

Data is contained within the article.
